# Radiological parameters of sagittal plane in children with cerebral palsy, walking or wandering

**DOI:** 10.1186/1748-7161-8-S1-P17

**Published:** 2013-06-03

**Authors:** JC Bernard, J Deceuninck, A Combey, E Berthonnaud

**Affiliations:** 1 Croix Rouge française CMCR des Massues, Lyon, France; 2 L’Hôpital Nord Ouest, Villefranche sur Saône, France; 3 Laboratoire de Physiologie de l’Exercice, Université de Lyon, Saint Etienne, France

## Background

The population of cerebral palsy (CP), walking or wandering, often has an abnormal profile clinically, unlike same aged adolescents without neuro-motor dysfunction.

## Aim

So, we wanted, in this work, to realize a radiological assessment of the static data in the spine-pelvis-thigh complex, in children with CP, and made a comparison with an asymptomatic population.

## Methods

The population of CP is made up 119 children, and the asymptomatic population made up of 652 children. The radiographs of the sagittal plane, in large format (30cmx90cm), are realized in a comfortable position, knees and hips in maximal extension. Analyses were performed, using the Optispine® software, to measure radiological parameters of the whole spine-pelvis-thigh.

## Results

Comparing the two populations, we found no difference in the shape parameter (pelvic incidence) for against a significant difference was demonstrated on the positional parameters (pelvic tilt and sacral slope) of the pelvis. Regarding the spine, we found a difference in the angulation of lumbar lordosis, and the orientation of the latter, as well as the number of vertebrae included in the kyphosis, and its orientation. There is also a significant difference in the C7 plumb line.

## Conclusion

We can say that the CP population is not specifically different from the control population. Growth disrupts the settings, with the need to prevent these troubles, as soon as possible, to the condition to be concerned, and able to search for.
